# Case Report: Identification and Functional Analysis of a Homozygous Synonymous Variant in the *PLOD1* Gene in a Chinese Neonatal With the Ehlers–Danlos Syndrome

**DOI:** 10.3389/fped.2022.813758

**Published:** 2022-02-17

**Authors:** Xiaodan Yan, Jianbo Shu, Yanyan Nie, Ying Zhang, Ping Wang, Weiwei Zhou, Xiaoyu Cui, Yang Liu

**Affiliations:** ^1^Department of Neonatology, Tianjin Children's Hospital (Tianjin University Children's Hospital), Tianjin, China; ^2^Graduate College of Tianjin Medical University, Tianjin, China; ^3^Tianjin Key Laboratory of Birth Defects for Prevention and Treatment, Tianjin, China; ^4^Tianjin Pediatric Research Institute, Tianjin Children's Hospital (Tianjin University Children's Hospital), Tianjin, China

**Keywords:** *PLOD1*, Ehlers-Danlos syndrome, newborn, synonymous variant, splicing

## Abstract

**Background:**

Kyphoscoliotic Ehlers–Danlos syndrome (kEDS; OMIM225400) is a rare autosomal recessive genetic disease caused by variants in the *PLOD1* gene. This research was conducted to verify the disease-causing gene in a Chinese neonatal family with the EDS.

**Methods:**

We recruited a Han Chinese neonate with *PLOD1*-related kEDS without kyphoscoliosis. Detailed clinical examination and laboratory tests were performed and whole exome sequencing (WES) was used to detect the pathogenic genes of the proband. *In vivo* experiments (reverse-transcription PCR, quantitative real-time PCR) and *in vitro* experiments (minigene analysis) were used to verify the function of variants suspected of affecting the splicing process. The effect of the splice site variant on the *PLOD1* transcript was analyzed using splice prediction programs NetGene2 and Alternative Splice Site Predictor (ASSP).

**Results:**

A homozygous synonymous variant c.1095C>T (p.Gly365, rs1032781250) in the *PLOD1* gene was found and verified in the family with kEDS. This splicing variant resulted in a premature termination codon of exon 10 and affected the expression of the four bases GCGC.

**Conclusion:**

Our research showed that the homozygous synonymous variant in *PLOD1* was the pathogenic cause in the proband. The combined application of WES and functional studies verified the effect of uncertain gene variants on splicing, upgrading pathogenicity evidence, and determining the cause of disease. This is helpful for the early diagnosis and treatment of kEDS.

## Introduction

Kyphoscoliotic Ehlers–Danlos syndrome (kEDS; OMIM 225400) is a rare autosomal recessive genetic disorder with an incidence of 1:100,000 that occurs early in life and mainly affects the musculoskeletal system (1). In 1998, the Villefranche etiology classified EDS into six major subtypes based on the severity of clinical symptoms, genetic patterns, and potential biochemical and molecular defects (2). According to the last classification of EDS, it is classified into 13 subtypes (3). kEDS is divided into two subtypes based on molecular pathology. The *PLOD1* variant causes kEDS-*PLOD1* and the *FKBP14* variant causes kEDS-*FKBP14*. Moreover, kEDS is characterized by severe intraocular hypotension at birth, progressive kyphosis, apparent skin hyper-elasticity, severe joint hyperactivity, and dislocation (4).

Most kEDS patients have a variant in the *PLOD1* gene, which encodes the collagen-modifying enzyme-lysine and 2-oxyglutarate-5-dioxygenase 1 (*PLOD1* or lysine hydroxylase 1) (3). Lysine hydroxylase 1 (LH1) is a posttranslational modification enzyme that plays an important role in the biosynthesis of collagen, it will through hydroxylation of helical lysyl residues in-Xaa-Lys-Gly-collagen sequences to hydroxy-lysyl residues, which serve as sites of attachment for carbohydrate units, and in the process of the formation of intra- and inter-molecular collagen cross-links (5). Lack of LH1 can lead to insufficient hydroxylation of collagen-dependent amino residues, thereby affecting cross-linking and making the affected tissue unstable (6). Therefore, the lack of this enzyme leads to the production of lysyl-pyridinoline (LP) and hydroxylysyl-pyridinoline (HP) in the body, which is then excreted abnormally *via* urine (7). The LH1 activity of skin fibroblasts in kEDS patients is 25% lower than that of normal people (8). To date, 56 variants have been reported in *PLOD1* according to the EDS variant database (https://eds.gene.le.ac.uk/home.php?select_db=PLOD1).

In this research, whole exome sequencing (WES) was used to analyze the genetic cause in a family with kEDS-*PLOD1*. We performed additional functional analyses for variants with uncertain significance (VUS) suspected of affecting splicing through *in vivo* experiments (reverse transcription PCR, quantitative real-time PCR) and *in vitro* experiments (minigene analysis) and gathered further pathogenic evidence. Finally, the results of WES combined with functional analysis strongly support the diagnosis of kEDS-*PLOD1* in the proband, who, to our knowledge, is the first neonatal Chinese kEDS-*PLOD1* patient without kyphoscoliosis. We hope our results will provide a solid foundation for genetic counseling and disease diagnosis.

## Materials and Methods

### Subjects

A Chinese family with kEDS-*PLOD1*, including three members, was recruited from Tianjin Children's Hospital in December 2020. This study has been approved by the ethics and human research committee of Tianjin Children's Hospital.

### DNA Extraction

We obtained about 4 mL of EDTA anticoagulant peripheral blood samples from the patient and his parents after the parents signed the informed consent form. Genomic DNA was extracted using the Blood Genomic DNA Mini kit (cat. no. CW0541; CoWin Biosciences) according to the standard procedures. We used the NanoDrop^®^ 2000 spectrophotometer (Thermo Fisher Scientific, Inc.) to assess the absorbance ratio between 260 and 280 nm by extracting 1 μl DNA. The measured concentrations of DNA were stored at −20°C.

### WES and Sanger Sequencing

The proband was analyzed by WES and Sanger sequencing was conducted on the whole family by KingMED (Tianjin, China). We use Chromas software (version 1.62; Technelysium Pty. Ltd.) to contrast the sequencing data with the target sequences in NCBI (https://www.ncbi.nlm.nih.gov/) to confirm the variant.

### Evaluating the Significance of the *PLOD1* Variants

To evaluate the significance of the *PLOD1* variants, we conducted reverse transcription PCR and quantitative real-time PCR (qPCR). Total RNA was extracted from lymphocytes and reverse transcribed with a reference operation by using the FastKing RT Kit (KR180123; Tiangen Biotech). The reverse transcription PCR reaction was followed by PCR amplification by using the forward primer (5′-CCTGGTCGGCGTGTTCATC-3′) and reverse primer (3′-TGCGGTAGCTGTCTAGGGAGAG-5′). The thermocycling conditions were: initial denaturation at 95°C for 2 min; followed by 35 cycles of 95°C for 30 s, 60°C for 30 s, and 72°C for 60 s; and a final extension step at 72°C for 5 min.

We then used BLAST (https://www.ncbi.nlm.nih.gov/tools/primer-blast/index.cgi) to design primers for quantitative real-time PCR containing the variant site, forward primer (5′-GAGGTGCGGATGGCGAAT-3′) and reverse primer (3′-GGGCAATGACGTTCTTGTTCT-5′). A normal patient cDNA was also used as a control. The cDNA was diluted 10 times before being add to each reaction. The appropriate qPCR conditions were: 95°C for 5 min followed by 45 cycles of 95°C for 10 s, 60°C for 10 s, 72°C for 10 s, and 40°C for 30 s (fluorescent dye solubilization were 95°C for 5 s, 65°C for 60 s, 97°C for 1 s for 1 cycle). This reaction was conducted on an LC480-II thermal cycler (Roche Diagnostics) by performing the melting curve analysis and recording the cycle threshold (Ct). We repeated the qPCR thrice and repeated each sample in quintuplicate to improve the error bars. All samples were run by GraphPad Prism software (version 8.0.1; GraphPad, USA). The mRNA expression level was calculated and normalized by using the ΔCt method relative to GAPDH.

### Minigene Constructions and Expression

**Figure 1:**
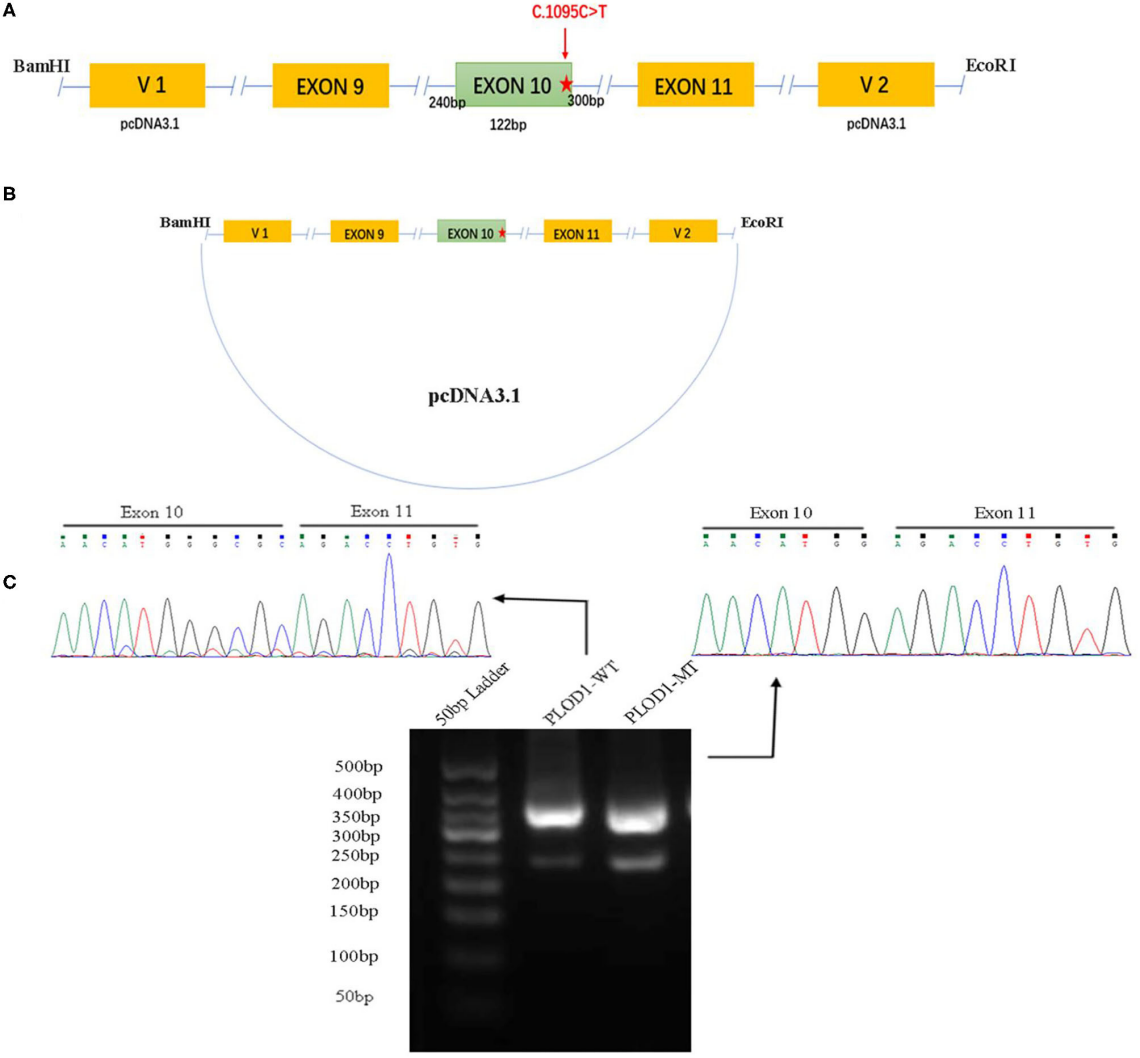
The minigene splicing assay based on the pcDNA3.1 exon trapping vector. **(A,B)** *PLOD1*-WT and *PLOD1*-MT plasmids containing exon 9–11 and flanking introns were separately cloned into the *Bam*HI and *Eco*RI cloning sites of the pcDNA3.1 vector (the red star, c.1095C>T). **(C)** Agarose gel electrophoresis of RT-PCR products. The electrophoresis results showed that the band of *PLOD1*-MT was lower than that of *PLOD1*-WT. The results of RT-PCR amplification of cDNA sequences generated by transfected 293T cells showed that c.1095C>T led to premature termination codon of exon 10 and affected the expression of the four bases GCGC.

To confirm the effect of the c.1095C>T variant, *in vitro* analysis was performed by using a minigene splicing assay based on the pcDNA3.1 expression vector (Figures 1A,B). With the ClonExpress Ultra One Step Cloning Kit (Vazyme Biotech Co., Ltd.), the fragments with the wild-type (WT) alleles involving 9–11 exon, flanked by approximately 120–300 nucleotides of upstream intronic sequence and downstream intronic sequence, were cloned into the splicing vector pcDNA3.1 using specific primers linking the *Bam*HI and *Eco*RI restriction enzyme sites (*Bam*HI: G^∧^GATCC; *Eco*RI: G^∧^AATTC). Primers were designed for each target fragment using CE Design software (version 1.04; Vazyme Biotech Co., Ltd.). The wild-type (WT) minigene plasmid was used as a template to generate the mutant-type (MT) minigene by using the Fast Site-directed Mutagenesis Kit (Tiangen Biotech [Beijing] Co., Ltd.). The wild and mutant type constructs were named *PLOD1*-WT and *PLOD1*-MT, respectively.

Both *PLOD1*-WT and *PLOD1*-MT were further transformed into *Escherichia coli* DH5α-competent cells (TaKaRa [Beijing] Co., Ltd.), confirmed by screening using conventional Sanger sequencing. Human epithelial kidney 293T (HEK 293T) cells were cultured in DMEM containing 10% fetal bovine serum (FBS), penicillin (100 U/L), and streptomycin (100 mg/L) at 37°C in a 5% CO_2_ atmosphere. One day before transfection, both cells were transferred to a 6-well culture plate to grow to approximately 70–80% confluence in an antibiotic-free medium. HEK 293T cells were then transfected with 2.5 μg plasmid DNA (*PLOD1*-WT, *PLOD1*-MT, and empty *PLOD1*-control) using OPTI-MEM^®^ IMedium and Lipofectamine 2000 (Invitrogen, Carlsbad, CA) according to the manufacturer's instructions. After 24 h, the cells were harvested and total RNA was extracted with Trizol reagent (Tiangen Biotech). Total RNA was reverse-transcribed to cDNA using FastKing RT Kit (with gDNase) (Tiangen Biotech) by using the following vector-specific primers: forward primer (*PLOD1-F*, 5′-GATGAAGCTCTGCCCACGGT-3′) and reverse primer (*PLOD1*-R, 3′-TTGTTCTGTTGGATCAGCAG-5′). Samples were heated to 95°C for 5 min, followed by 34 cycles of DNA denaturation (95°C for 60 s), annealing (58°C for 30 s), and polymerization (72°C for 30 s). After the last cycle, the samples were incubated for an additional 5 min at 72°C. The PCR products were subsequently separated by electrophoresis on a 2.5% agarose gel, and the intensity of each band was quantified by ImageJ software. All transcripts were analyzed by sequencing.

## Results

### Subjects

**Figure 2:**
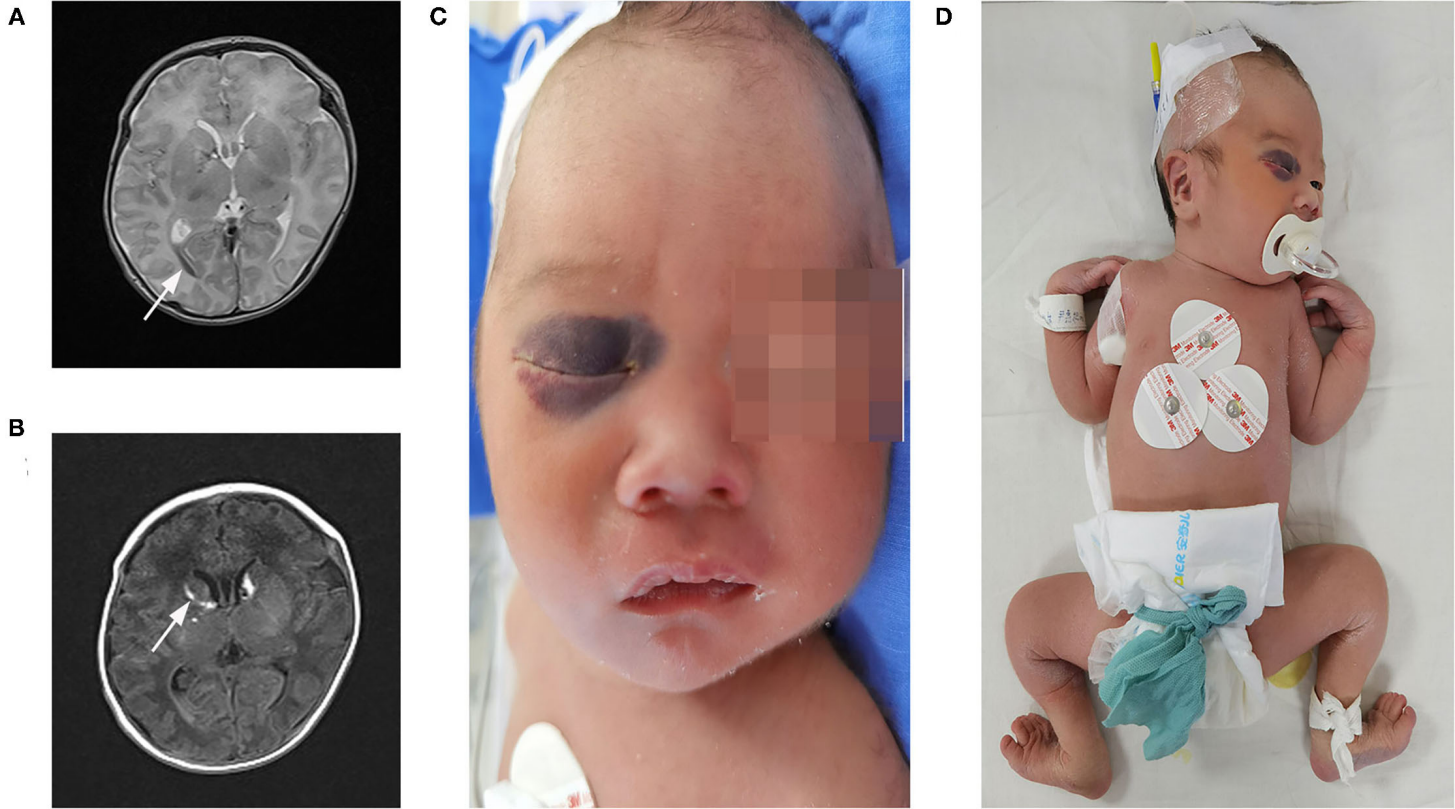
Clinical features and imaging manifestations of proband. **(A,B)** Cranial MRI: intraventricular hemorrhage and subarachnoid hemorrhage (direction of arrow). **(C)** Facial bruises. **(D)** Wrist, excessive abduction of the bilateral hip joints, bipedal valgus, and excessive flexion.

The patient was a 22-h-old male neonate and the second child of unrelated Chinese parents. He was born at 36^+3^ weeks of gestation through vaginal delivery, and the mother had a history of unknown spontaneous abortion at 19 weeks of pregnancy. The proband weighed 2,800 g at birth and measured 48 cm in length and had a head circumference of 34.5 cm, with a normal Apgar score. Physical examination revealed no kyphoscoliosis, wrist sagging, excessive abduction of the bilateral hip joints, bipedal valgus and excessive flexion, muscle weakness, and swelling of hands and feet. The patient's primitive reflexes could not be elicited, and the mental response was weak. The patient's cranial magnetic resonance imaging (MRI) showed intraventricular hemorrhage and subarachnoid hemorrhage (Figures 2A,B). Facial examination showed bruises on the right eye socket, ears, and both sides of the nose, a 3 × 3-cm fluctuating mass on the top of the head, and a slightly higher palatal arch (Figures 2C,D). Ambulatory electroencephalography (AEEG) was abnormal, with increased trace discontinue (TD) and prolonged interburst interval (IBI). Echocardiography showed patent ductus arteriosus and patent foramen ovale. X-ray showed that sclerotin was normal and the patient did not have thoracic scoliosis on chest X-Ray. Electrophysiological examination revealed multiple peripheral nerve lesions, which improved after 13 days but still showed multiple damages. The serum creatine kinase (CK) level was 8505 U/L (normal, <310 U/L). CK-MB was 265 U/L (normal, <24 U/L). CK-MBmass was 202.9 ng/mL (normal, <14.97 ng/mL). These results suggested that the patient had myocardial damage. The Thyroid Stimulating Hormone (TSH) level was 7.377 mIU/L (normal, <5.33 mIU/L) and 25-hydroxyvitamin D level was 5.9 ng/mL (normal, >15 ng/mL). Routine biochemical examination, acute phase reactants, blood and sputum culture results, neonatal blood and urine screening, blood gas analysis, and levels of lactic acid and serum immunoglobulin were normal.

### Detection and Certification of the Variants

**Figure 3:**
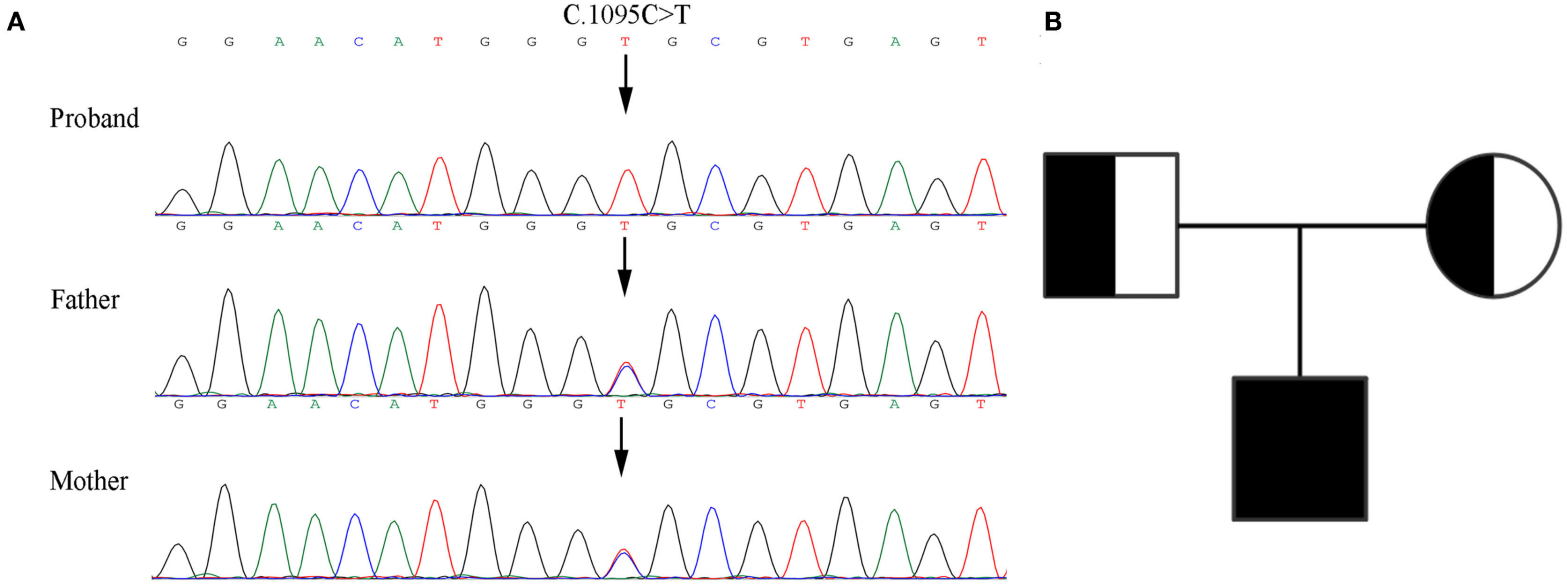
Genetic analysis of the pedigree. **(A)** Sanger sequencing of the family with kEDS -*PLOD1* shows that the proband is homozygous and the parents are heterozygous. **(B)** Pedigree chart.

The patient's WES showed that there was a homozygous synonymous variant on exon 10 of *PLOD1* (NM_000302.3), c.1095C>T (p.Gly365=). Based on the result of WES, we performed Sanger sequencing on the parents and the proband. The parents were all heterozygous carriers, which was in accordance with the autosomal recessive inheritance pattern (Figures 3A,B).

### Prediction of Pathogenicity

**Figure 4:**
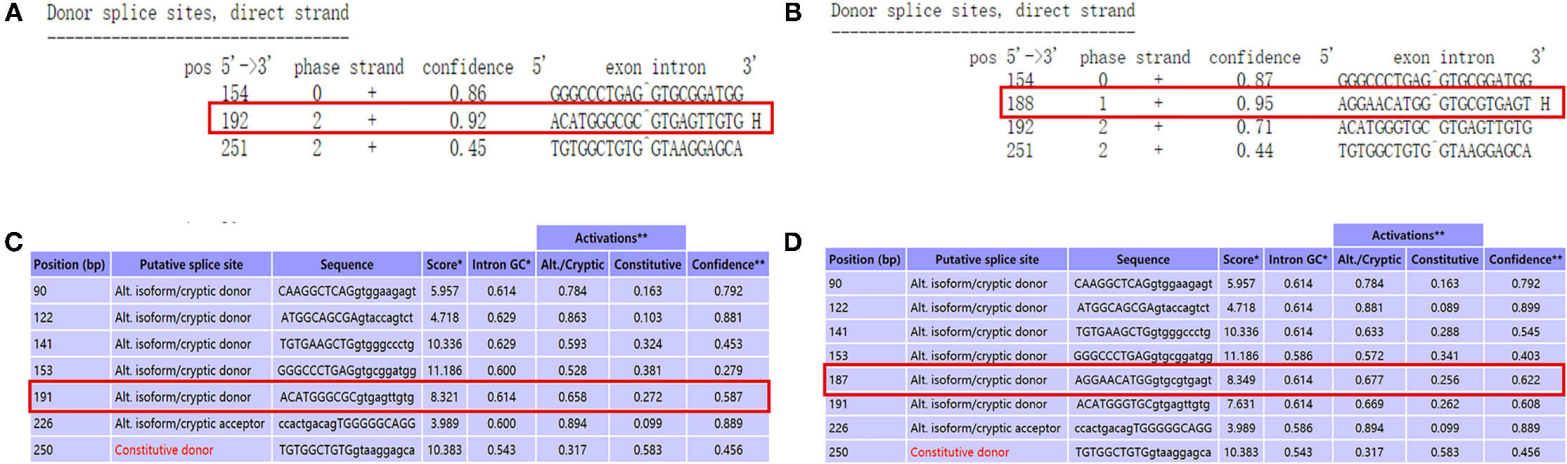
Use prediction software to analyze the variant site. **(A)** The predict result of wild type by using NetGene2 Server (the red square represented). **(B)** “AGGAACATGG GTGCGTGAGT” was the appropriate predict result to affects the splicing impact by using NetGene2 Server (the red square represented). **(C)** The predict result of wild type by using ASSP (the red square represented). **(D)** The predict result of mutant type by using ASSP (the red square represented).

We used NetGene2 Server (http://www.cbs.dtu.dk/services/NetGene2/) to predict the c.1095C>T (p.Gly365=) variation that affects the splicing impact. The result was consistent with the pathogenicity site, which caused the premature termination codon of the exon, and the confidence score of the result was 0.95 (Figures 4A,B). We also used the Alternative Splice Site Predictor (ASSP) (http://wangcomputing.com/assp/index.html) to predict the variation, which conformed to the NetGene2 Server (Figures 4C,D).

### Variant Validation and Analysis

**Figure 5:**
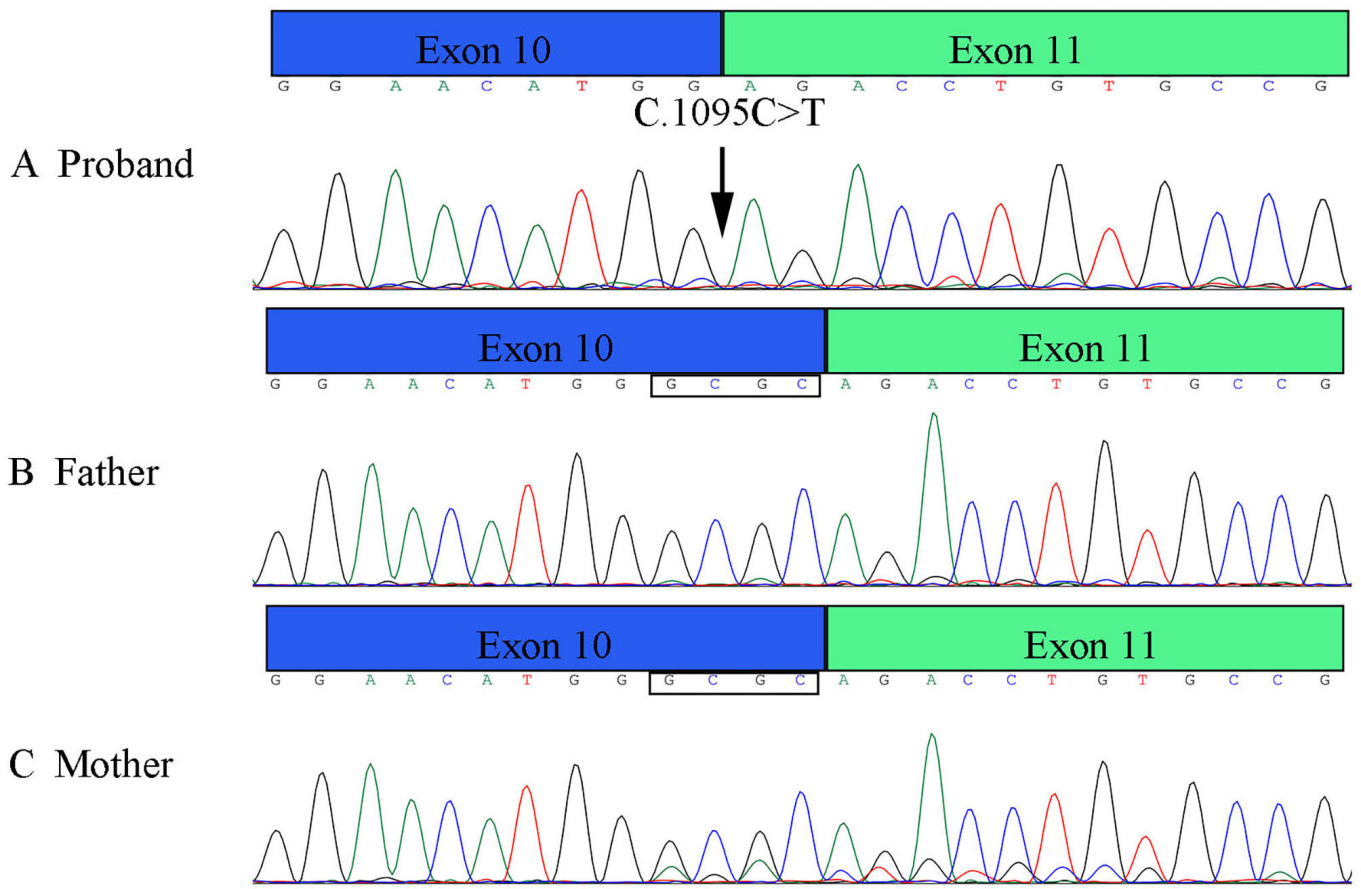
Results of RT-PCR of *PLOD1* variant in pedigree. **(A)** The proband variant resulted in a premature termination codon of exon 10 and affected the expression of the four bases GCGC. **(B,C)** Parents' cDNA at exon 10 and exon 11.

RT-PCR verified that the variant affects the splicing, which caused the premature termination codon of exon 10 to affect the expression of the four bases of GCGC (Figure 5). These results are consistent with those of Sanger sequencing, NetGene2 Server, and ASSP.

**Figure 6:**
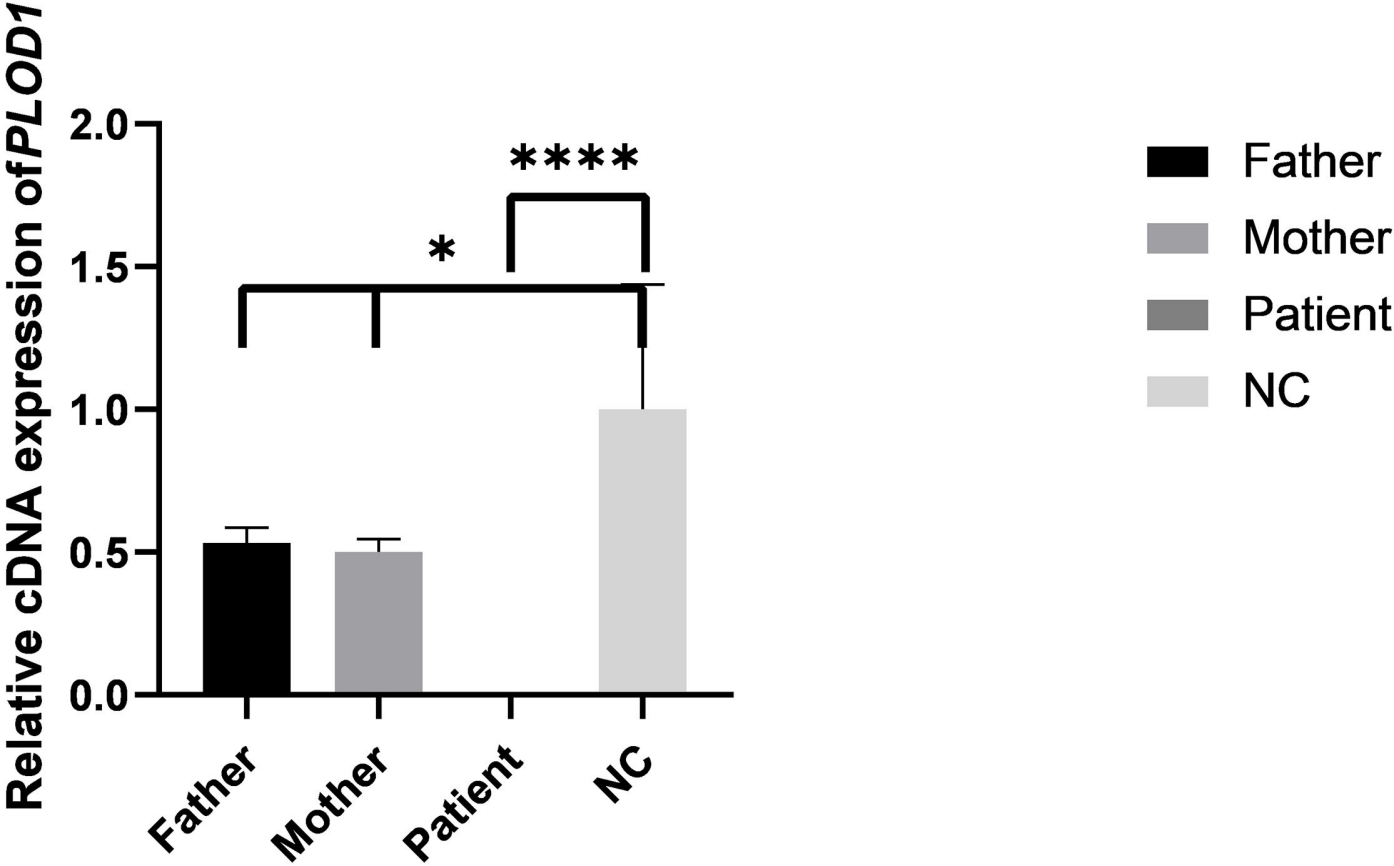
The results of qPCR in pedigree and normal control (NC). Difference of qPCR expression levels in Father, Mother, Patient, and NC (*P < 0.05, ****P < 0.0001).

The results of the qPCR revealed that the mRNA level of the proband was significantly lower than the normal control (NC) (Figure 6). The mRNA levels of parents were both half of the normal control, but both parents were phenotypically normal. These results collectively confirmed that the premature termination codon of exon 10 led to abnormal transcription of *PLOD1* and caused disease.

### Minigene Analysis

The *PLOD1*-WT and *PLOD1*-MT minigene constructs were validated in 293T cells, where they produced the expected transcripts. The electrophoresis results showed that the amplified cDNA band corresponding to the spliced transcripts in the *PLOD1*-MT was lower than that in the *PLOD1*-WT (Figure 1C). In other words, the *PLOD1*-MT PCR produced a smaller cDNA product than the *PLOD1*-WT. This is likely because the expression of *PLOD1*-MT minigene led to a premature termination codon of exon 10, which was verified by the sequence of cDNA-amplified products. It proved that the c.1095C>T variant was the cause of premature termination codon of exon 10.

### Genetic Variation Analysis of *PLOD1* in the Patient

The pathogenicity of *PLOD1* variants in the proband was assessed based on the American College of Medical Genetics and Genomics (ACMG) standards and guidelines for the interpretation of sequence variants (9). The c.1095C>T variant is absent from controls in the Exome Sequencing Project, 1000 Genomes Project, or Exome Aggregation Consortium (PM2). *In vitro* or *in vivo* functional studies support a damaging effect on the gene (PS3). According to the splice prediction program, the c.1095C>T variant significantly affects splicing (PP3). The patient's phenotype or family history was highly specific for kEDS (PP4). As a result, the c.1095C>T variant was classified as “likely pathogenic” based on the above evidence.

## Discussion

kEDS-*PLOD1* is a rare autosomal recessive genetic disorder. According to the latest revision of the international standards of EDS, there are three main standards in the diagnosis of kEDS-*PLOD1* including congenital muscle weakness and congenital or early-onset of kyphosis (progressive or non-progressive); accompanied by dislocation or subluxation (especially in the shoulders, knees, and hips) (3). There are 10 secondary standards: skin excessive extension; simply bruisable skin; medium-sized artery rupture or aneurysm; osteopenia or osteoporosis; blue sclera; hernia (navel or groin); chest deformity; talipes equinovarus; marfanoid habitus; and refractive errors (myopia or hyperopia). In addition, there are also gene-specific secondary criteria for *PLOD1* including skin brittleness (easy bruising, skin fragility, poor wound healing, widening atrophic scars); sclera, and eye brittleness or rupture; microcorneas; and facial deformities. Patients are required to meet the first two main standards and the third main standard or three secondary standards for a confirmed diagnosis of kEDS-*PLOD1* (7). The proband met the two main standards, including congenital muscle weakness; congenital or early-onset kyphosis (progressive or non-progressive); and the three secondary standards including skin excessive extension, simply bruisable skin, and skin brittleness. Especially the genetic detection of *PLOD1* variants and kEDS could be diagnosed. As per the latest kEDS-*PLOD1* diagnostic criteria of 2017, patients like our proband with kEDS-*PLOD1* do not necessarily have kyphosis at birth and infancy (3). According to previous reports, patients will have more complications during their development, such as recurrent pneumonia and cardiac failure (caused by severe kyphoscoliosis), chronic respiratory failure, glaucoma, retinal detachment, arteriorrhexis, arterial aneurysm, and especially circulatory system complications that can be more life-threatening (2, 10).

To improve the long-term survival rate of kEDS-*PLOD1* and the quality of patients' life, early diagnosis and treatment for patients, along with improving the prognosis and long-term follow-up are crucial. On the basis of previous reports of vascular ruptures, it could be seen more commonly in kEDS*-PLOD1*. Therefore, we need to take measures to improve the growth and development of blood vessels. For example, regular echocardiographic examinations are used to assess the cardiovascular condition (computed tomography and angiography may be considered if the disease changes further), monitor the size of the aortic root until the patient is 5 years old, and assesses the condition of mitral or tricuspid valve prolapse (11). It is worth noting that the neurological signs and neurodevelopmental intelligence of affected children are generally normal, but learning disabilities have been reported in eight patients (two of whom had reported prenatal or perinatal intracranial hemorrhage) (1, 12). The proband also had intracranial hemorrhage and unexplained ecchymosis on the face, hands, and feet. However, the proband was delivered normally, which does not support the performance of petechial and intracranial hemorrhage caused by birth injury. Further examination of the patient's blood coagulation function level showed normal results, also not supporting the intracranial hemorrhage. In summary, intracranial hemorrhage is related to vascular disease or arterial rupture unique to kEDS-*PLOD1*. At present, the follow-up of proband shows that the muscle strength remains poor and the absorption of head hematoma improves. We suggest that in newborns with joint hypermobility, with or without kyphoscoliosis, low muscle strength, skin fragility, and congenital low intraocular pressure, clinicians should highly consider kEDS-*PLOD1*.

*PLOD1* is located on chromosome 1p36.22 and contains 20 exons. The pathogenic variant in *PLOD1* causes kEDS-*PLOD1*. The *PLOD1* gene encodes LH1 and plays an important role in maintaining the stability of intermolecular crosslinking. The LH1 activity of fibroblasts in patients is lower than that in normal people. According to previous reports, 15% *PLOD*1^−/−^ mice died within 1–4 months because of the degeneration of collagen fibers and abnormal smooth muscle cells in the tissues of living mice, indicating that the mice developed aortic dissection and hence aging and rupture of vascular function (13). In the past reports of the *PLOD1* variant, the most common was large duplication of the 10–16 exons that was >20% in kEDS-*PLOD1* patients, and the others were point variants, insertions, and deletions that caused premature termination of codons and splicing variants caused exon skipping (14). We analyze the characteristics of a genetic and pathogenic variant in *PLOD1*.

In this research, we verified in detail the genetic information of c.1095C>T (p. Gly365=) in *PLOD1* in a Chinese neonatal pedigree. In this pedigree, the proband had a homozygous variant, and the parents were all heterozygous carriers. The c.1095C>T synonymous variant in *PLOD1* introduced a single nucleotide transition from C to T at nucleotide 1095. Based on the ACMG standards and guidelines for the interpretation of sequence variants, c.1095C>T is classified as “VUS” because of insufficient evidence. The most canonical splicing-site variants are located at the ±1, 2 positions of the 5′ or 3′ splice sites (15). Therefore, the pathogenicity of the c.1095C>T cryptic splice site variant is still unclear. In order to verify the pathogenic evidence that VUS (c.1095C>T) may affect splicing, we conducted a minigene assay on the splicing of *PLOD1*. Thus, the premature termination codon of exon 10 was confirmed. This conclusion was also confirmed in RT-PCR. As shown by the qPCR results, the synonymous variant affected the splicing of exons, and the mRNA level of the proband was significantly lower than that of the normal control. The mRNA levels of both parents were half of the normal control, but they were phenotypically normal, which might not be high enough to make the parents sick. Hence, the pathogenicity threshold of the disease could be further explored. Interestingly, before *in vitro* and *in vivo* experiments, we applied the software to predict the splicing impact and also reached consistent conclusions. An increasing number of research studies have reported that synonymous variants without changing the amino acid sequence could also become dangerous factors for disease (16–18). Variants leading to abnormal splicing of mRNA are understood to be the main pathogenic factors (17). It is estimated that 90% of disease-related synonymous variants may affect splicing function (19). Recently, the Gene Panel and WES have significantly increased the diagnostic positive rate of rare diseases, enabling genetic diagnosis in 35–50% cases, and in particular, the application of RNA-seq to the diagnosis of genetic diseases has developed considerably (20).

This variant c.1095C>T (p.Gly365=) has already been named as rs1032781250 (1). In that case report, a 9-year-old male patient had the c.1095C>T mutation, and the authors hypothesized that the variant caused abnormal splicing of exon 10, which terminated the translation prematurely. However, our study carried out a more comprehensive genetic inspection and verification and described the genetic pathogenic mechanism of splicing on kEDS*-PLOD1*. The 9-year-old male patient was born with kyphoscoliosis, but he did not get a clear kEDS diagnosis during the neonatal period or the long-term follow-up, and still could not walk independently. Nevertheless, the proband in our study was born without kyphoscoliosis. We followed up the proband until the time of writing this manuscript and can confirm that the patient has still not developed kyphoscoliosis. We suspect that patients who are not born with kyphoscoliosis will develop this later in life. Therefore, we suggest that when kyphoscoliosis is detected in the neonatal period, relevant auxiliary examinations and genetic testing should be carried out promptly to exclude the differential diagnosis and ultimately improve it to increase the early diagnosis rate of kEDS-*PLOD1*.

In conclusion, our research verified that the homozygous synonymous variant c.1095 C>T (p.Gly365=) in the *PLOD1* gene caused the pathogenic phenotype in the proband. The combined application of WES and functional studies contribute to verifying the effect of uncertain gene variants on splicing, upgrading pathogenicity evidence, and determining the cause of disease. This is helpful for the early diagnosis and treatment of kEDS.

## Data Availability Statement

The datasets for this article are not publicly available due to concerns regarding participant/patient anonymity. Requests to access the datasets should be directed to the corresponding author.

## Ethics Statement

The studies involving human participants were reviewed and approved by the Ethics and Human Committee of Tianjin Children's Hospital. The patients provided written informed consent to participate in this study.

## Author Contributions

XY and JS conceived the idea, conceptualized the study, and drafted the manuscript. YN and YZ collected the data. PW and WZ analyzed the data. JS and YL reviewed the manuscript. All authors read and approved the final draft.

## Funding

This research was funded by the Program of Tianjin Science and Technology Plan (18ZXDBSY00170) and the Project of Tianjin Health Science and Technology (ZC20120 and KJ20166).

## Conflict of Interest

The authors declare that the research was conducted in the absence of any commercial or financial relationships that could be construed as a potential conflict of interest.

## Publisher's Note

All claims expressed in this article are solely those of the authors and do not necessarily represent those of their affiliated organizations, or those of the publisher, the editors and the reviewers. Any product that may be evaluated in this article, or claim that may be made by its manufacturer, is not guaranteed or endorsed by the publisher.
